# Primary synchronous colloid adenocarcinoma and squamous cell carcinoma in the same lung: A rare case report

**DOI:** 10.1097/MD.0000000000024700

**Published:** 2021-02-12

**Authors:** Yang Liu, Lin Kang, Han Hao, Xiuzhi Zhang, Guona Zheng, Xiaowan Guo, Huanfen Zhao

**Affiliations:** a Department of Pathology, Hebei General Hospital; b Department of Pharmacology, Hebei Medical University; c Department of Image, Hebei General Hospital, Shijiazhuang, China.

**Keywords:** colloid adenocarcinoma, diagnosis, double primary lung cancer, squamous cell carcinoma

## Abstract

**Rationale:**

Double primary lung cancer (DPLC) is a relatively rare type of lung cancers. According to whether the diagnosis interval between lesions is more than 6 months, it can be divided into synchronous DPLC (sDPLC) and metachronous DPLC (mDPLC). Here, we describe a case of sDPLC in which one of the components is a rare colloid adenocarcinoma (CA).

**Patient concerns:**

A 69-year-old male was admitted to the hospital due to chest distress and shortness of breath for 1 year, getting worse in the last 15 days.

**Diagnosis:**

Both HE staining and IHC supported the diagnosis of CA in the right lower lobe and moderately differentiated squamous cell carcinoma in the right upper lobe.

**Interventions:**

The patient was treated with 3 cycles of adjuvant chemotherapy with pemetrexed and lobaplatin after the right upper lobectomy, wedge resection of the right lower lobe and lymph node dissection under video-assisted thoracoscope.

**Outcomes:**

Our plan was to follow him up with general physical examination, chest-abdomen CT and serum tumor markers every 6 months for 2 years. The patient was still alive until the last follow-up in November 2020.

**Lessons:**

CA of the lung is a rare primary lung adenocarcinoma. The diagnosis should be based on the patient's clinical characteristics, imaging examination and pathological characteristics, and also need to be differentiated from other mucinous adenocarcinomas. Interestingly, our patient developed not only a CA in the right lower lobe, but also a moderately differentiated squamous cell carcinoma in the right upper lobe.

## 1 Introduction

Multiple primary lung cancer (MPLC) is a relatively rare type of lung cancers, while double primary lung cancer (DPLC) is the most common type in MPLCs.^[1–3]^ DPLC is defined as the occurrence of 2 primary malignant tumors simultaneously or successively in the patient's lungs.^[4]^ According to whether the diagnosis interval between lesions is more than 6 months, it can be divided into synchronous DPLC (sDPLC) and metachronous DPLC (mDPLC). With the continuous progress of clinical diagnosis technology, the detection rate of multiple pulmonary nodules increases year by year, and the diagnosis rate of MPLC also presents an increasing trend.^[5]^

Pulmonary colloid adenocarcinoma (CA) is also 1 rare tumor, accounting for about 0.24% of all primary lung cancers.^[6]^ This tumor is considered to be an adenocarcinoma with rich mucus that fills the alveolar cavity and destroys the alveolar walls. CA had always been diagnosed as “mucinous cystadenoma”, “mucinous cystic tumor” or “mucinous cystadenocarcinoma” before, which was classified as a special type of adenocarcinoma by WHO (2015) - Lung tumor classification.^[7,8]^ As CA is rare in clinical practice, only a few cases have been reported. Here, we described an interesting case of CA that coexists with squamous cell carcinoma in the same lung.

## 2 Case presentation

**Figure 1:**
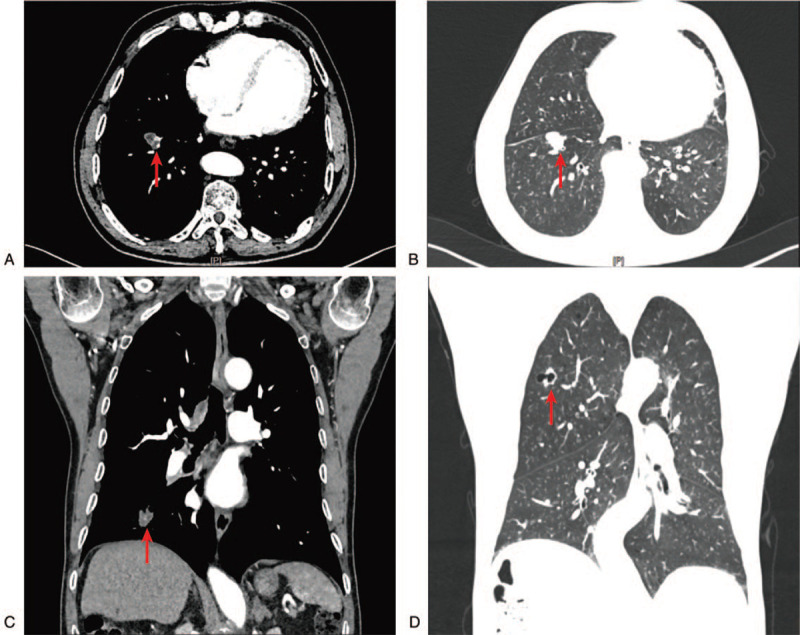
Chest CT. A-C, In the right lower lobe (S_8_), 1 nodule measured 20 × 15 × 16 mm showed clear boundary, lobulated margin, and multiple hive-like low density areas in the center (arrows). D, In the right upper lobe (S_1_), another nodule measured 20 × 14 × 13 mm showed irregular margin, uneven thickness of cavity wall, and local nodular solid density shadow (arrow). CT = computed tomography.

In July 2018, a 69-year-old male was admitted to the hospital due to chest distress and shortness of breath for 1 year, getting worse in the last 15 days. Physical examination after admission showed no abnormality. The patient had a smoking history of more than 50 years, but no peripheral vascular disease, no previous history of chronic diseases such as hypertension, coronary heart disease and diabetes, no infectious history of hepatitis, typhoid fever and tuberculosis, no history of trauma and no family hereditary disease. Contrast-enhanced CT of the chest revealed one nodule measured 20 × 15 × 16 mm in the right lower lobe (S_8_) had clear boundary, lobulated margin, slight enhancement, and multiple hive-like low density areas in the center (Fig. 1A-C). In addition, the image showed another nodule measured 20 × 14 × 13 mm had irregular margin, uneven thickness of cavity wall, and local nodular solid density shadow in the right upper lobe (S_1_, Fig. 1D). Ultrasonography of liver, gallbladder, spleen, pancreas, and kidney showed no abnormality. The tumor marker carcino-embryonic antigen was 5.16 ng/mL; cytokerantin-19-fragment was 3.54 ng/mL; neuronspecific enolase was 13.00 ng/mL.

After all preoperative examinations were completed, wedge resection of the upper and lower lobes in the right lung was performed under video-assisted thoracoscope according to the locations of the nodules. Based on the result of frozen pathology, 1 nodule with a maximum diameter of 2.0 cm was observed in the wedge tissue of the right lower lobe, which showed solid section, greyish-white, local colloidal substance and clear boundary with surrounding tissue. Another nodule with a maximum diameter of 1.8 cm was found in the wedge tissue of the right upper lobe. It showed solid section, greyish-white and unclear boundary with surrounding tissue. Furthermore, the patient underwent the right upper lobectomy and lymph node dissection under video-assisted thoracoscope.

**Figure 2:**
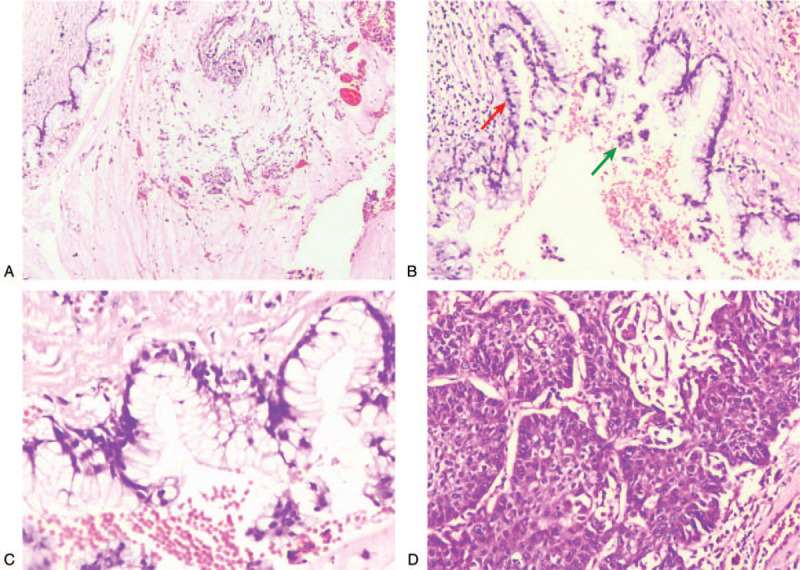
Hematoxylin-eosin (HE) staining. A-C, HE staining of the S_8_ nodule. A, Tumor tissues showed rupture of alveolar septum, and rich in mucous which formed mucous pools. HE × 40. B, Some tumor cells displayed as monolayer columnar cells lined by the thickened fibrous alveolar walls (red arrow). Other tumor cells displayed as clustered or micro-papillary morphology floating in the mucous pools (green arrow). HE × 100. C, Tumor cells were light to moderate atypia. HE × 200. (D) Squamous cell carcinoma nests were be found in the S_1_ nodule. HE × 200.

Hematoxylin-Eosin (HE) staining revealed that tumor tissues, in the S_8_ nodule, showed rupture of alveolar septum, and rich in mucous which formed mucous pools. The alveolar cavity was also filled with a large amount of mucous. Some tumor cells, whose nuclei were located in the basement and cytoplasm was filled with mucous, displayed as monolayer columnar cells lined by the thickened fibrous alveolar walls. Other tumor cells, which were light to moderate atypia, displayed as clustered or micro-papillary morphology floating in the mucous pools (Fig. 2A-C). Furthermore, in the S_1_ nodule, the tumor cells were distributed in nests of varying sizes, and part of cancer cells was growing in alveolar cavities under low magnification. High magnification showed that keratin pearls were observed in the centers of cancer nests, and intercellular bridges were detected between the tumor cells (Fig. 2D).

**Figure 3:**
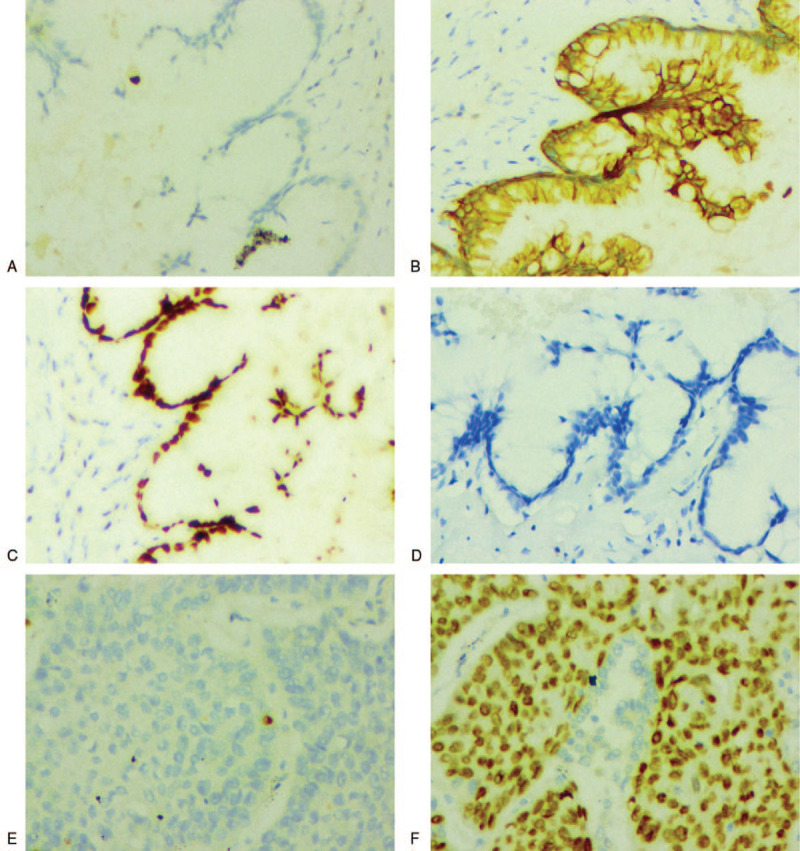
Immunohistochemistry (IHC) staining. A-D, IHC of the S_8_ nodule. A, TTF-1; B, CK20; C, CDX2; D, P40. E-F, IHC of the S_1_ nodule. E, TTF-1; F, P40. IHC × 200.

Immunohistochemically, the nodule in the right S_8_ was negative for TTF-1, NapinA, P40 and CK5/6, but with the positive expression of CK7, CK20, CDX2, MUC2; the nodule in the right S_1_ was positive for P63, P40, CK5/6 and negative for CK7, TTF-1, NapinA (Fig. 3). Therefore, both HE staining and immunohistochemistry (IHC) supported the diagnosis of CA (T1bN0M0, stage IA2) in the lower lobe and moderately differentiated squamous cell carcinoma (T1bN0M0, stage IA2) in the upper lobe. The patient was discharged 11 days after surgery. Three cycles of adjuvant chemotherapy with pemetrexed and lobaplatin were performed postoperatively. Our plan was to follow him up with general physical examination, chest-abdomen CT and serum tumor markers every 6 months for 2 years. The patient was still alive until the last follow-up in November 2020.

## 3 Discussion

Multiple primary lung cancer (MPLC) was first reported by Beyreuther in 1924.^[1]^ MPLC was considered to be a rare disease in the past, but according to research reports in recent years, its incidence is on the rise. Synchronous multiple primary lung cancer (sMPLC) is defined as 2 lung malignancies diagnosed within 6 months of each other. Guo et al^[9]^ (2017) summarized the clinicopathological characteristics and prognosis of 357 cases of sMPLC. There are 269 patients with double primary lung cancer (DPLC), 65 patients with triple primary lung cancer (TPLC) and 23 patients with 4 or more primary lung cancer. sMPLC was more common in males than females, and the average age of onset was about 60 years. Lesions (68.55%) mainly occurred in the upper lobe, especially the right upper lobe. The most common pathologic pattern was adenocarcinoma and adenocarcinoma (92.16%), followed by adenocarcinoma and squamous cell carcinoma (3.08%). Lobectomy and systemic lymph node dissection was recommended for sMPLC with lesions larger than 2 cm. Local resection was recommended for small lesions. Postoperative chemotherapy or targeted therapy should be used when necessary. The 3-year overall survival and 5-year overall survival in the 357 cases were up to respective 91.93% and 84.37%. Smoking history, the diameter of the maximum lesion, lymph node metastasis and pleural invasion were the independent risk factors for prognosis. Early diagnosis and positive treatment could improve the prognosis and survival of patients. Here, we describe 1 case of new synchronous DPLC combination of colloid adenocarcinoma (CA) and squamous cell carcinoma, which had never been reported.

Pulmonary CA is an extremely rare subtype of lung adenocarcinomas. As reported in the literature, the age of the pulmonary CA patients ranged from 32.0 to 81.5, and the mean age was 57.0 to 69.7. The ratio of male to female ranged from 5:19 to 7:3. The occurrence of each lung was more common in the right lung than in the left lung. The tumor diameter was 0.5–15.0 cm.^[7,10,11]^ CT mostly showed cystic density shadow which was almost located at the center of the CA nodule, with mild annular enhancement or no enhancement.^[6,12]^ This case is a 69-year-old male patient with a smoking history of more than 50 years. The CA measured maximum diameter 2.0 cm occurs in the lower lobe of the right lung. CT findings of CA showed lobulated and slightly low-density shadow, but no obvious enhancement. The above case description is consistent with the literature reported.

Histologic morphology of pulmonary CA requires differential diagnosis from other diseases.

1. solid predominant lung adenocarcinoma with mucous secretion^[13]^: the solid nests of tumor cells are predominant, and a few cells are filled with a small amount of secrete mucus;
2. invasive mucinous adenocarcinoma^[14]^: the tumor cells are mainly goblet-shaped or columnar, adherent to the wall, showing multifocal and leaping growth. The alveolar structure still exists. While CA is characterized by the destruction of alveolar walls via large amounts of mucus secreted by tumor cells that float in a mucous lake;
3. bronchial adenoma^[15]^: the alveolar cavity is filled with mucous, and the tumor tissue has an glandular or papillary structure. However, basal cell immunohistochemical markers P63 and P40 are positive for bronchial adenoma, which can be differentiated from pulmonary CA;
4. mucinous adenocarcinoma in gastrointestinal tract, breast, skin or other places can metastasize to the lungs,^[16–18]^ and it is difficult to differentiate the morphology from the primary mucinous adenocarcinoma of the lung. The clinical history and imaging examination of the patient are of great value in differentiating the metastatic mucinous adenocarcinoma from the primary pulmonary CA.

Immunohistochemically, the expression rates of TTF-1 and NapsinA were low in primary pulmonary CA (33% and 17% positive rates, respectively), while CK20, CDX2, and MUC2 were highly expressed.^[6]^ TTF-1 and NapsinA were often positively expressed in solid predominant lung adenocarcinoma with mucous secretion.^[13,19]^ CK20, CDX2, MUC2, and Villin were often negative in the invasive mucinous adenocarcinoma.^[19,20]^ However, metastatic mucinous adenocarcinoma of the gastrointestinal tract was positive for SATB2 and negative for CK7.^[19,21]^ In this case, the right S_8_ nodule is positive expression in CK7, CK20, CDX2, and MUC2, but negative expression in TTF-1 and NapsinA. So the immunophenotype is also consistent with pulmonary CA, and there is no history of gastrointestinal tract.

In summary, CA of the lung is a rare primary lung adenocarcinoma. The diagnosis should be based on the patient's clinical characteristics, imaging examination and pathological characteristics, and also need to be differentiated from other mucinous adenocarcinomas. Interestingly, this patient developed not only a CA in the right lower lobe, but also a moderately differentiated squamous cell carcinoma in the right upper lobe.

## Acknowledgments

The authors thank the patient who provided informed consent for publication of the case.

## Author contributions

**Data curation:** Yang Liu, Lin Kang, Han Hao, Xiuzhi Zhang, Guona Zheng, Xiaowan Guo, Huanfen Zhao.

**Formal analysis:** Yang Liu.

**Investigation:** Yang Liu, Lin Kang, Xiuzhi Zhang, Huanfen Zhao.

**Methodology:** Yang Liu, Lin Kang, Han Hao.

**Project administration:** Huanfen Zhao.

**Writing – original draft:** Yang Liu, Huanfen Zhao.

**Writing – review & editing:** Huanfen Zhao.

## Abbreviations

Abbreviations: CA = colloid adenocarcinoma, CT = computed tomography, DPLC = double primary lung cancer, HE = hematoxylin and eosin, IHC = immunohistochemistry, MPLC = multiple primary lung cancer.
